# Photovoice, emotional health, and structural inequalities: adolescent voices from an intercultural neighborhood in the global south

**DOI:** 10.3389/fpubh.2025.1709042

**Published:** 2026-01-23

**Authors:** Matías Araya-Tessini, María Sol Anigstein Vidal, Alicia Arias-Schreiber Muñoz, Constanza Jacques-Aviñó

**Affiliations:** 1 Departamento de Antropología, Universidad de Chile, Santiago, Chile; 2 Escuela de Salud Pública, Facultad de Medicina, Universidad de Chile, Santiago, Chile; 3 Departamento de Salud y Pueblos Indígenas e Interculturalidad, Ministerio de Salud, Chile; 4 Unitat Transversal de Recerca. Fundació Institut Universitari per a la Recerca a l'Atenció Primària de Salut Jordi Gol i Gurina (IDIAPJGol), Barcelona, Spain; 5 Network for Research on Chronicity, Primary Care and Health Promotion (RICAPPS), Barcelona, Spain; 6 Universitat Autònoma de Barcelona (UAB), Bellaterra, Spain

**Keywords:** adolescence, collective health, emotional health, global south, photovoice, participatory research, social inequalities, intersectionality

## Abstract

**Introduction:**

The research is framed within the Latin American collective health perspective, which understands emotional well-being as a relational and situated phenomenon shaped by social determinants. We aimed explores how a group of cisgender and non-binary adolescent girls from Global South construct and give meaning to their emotional well-being in a context marked by structural inequalities, migration, and cultural diversity.

**Method:**

Using the Photovoice method, a participatory strategy was developed that enabled participants to express their emotional experiences through images and collective narratives. Intersectionality and adolescent agency were central to the analysis. The study was conducted between 2022 and 2023 in a single-sex public school located in an intercultural neighborhood of Santiago, Chile, during the return to in-person learning after 2 years of COVID-19 lockdown.

**Results:**

Findings show that emotional well-being is linked to both intimate spaces (e.g., solitude, bonds with companion animals) and collective ones (e.g., friendships, family, activism, and school). Additionally, unpleasant emotions, such as desolation, were interpreted as part of emotional development. Nature emerged symbolically to reflect complex emotions and as a source of ecological concern.

**Conclusions:**

The study underscores the importance of creating spaces for participation, listening, and recognition, and calls for public policies that actively incorporate adolescent voices in shaping emotional of well-being.

## Introduction

The emotional well-being of adolescents has been profoundly affected in recent years, especially after the COVID-19 syndemic—understood as a health crisis intertwined with social, economic, and territorial inequalities. The collapse of daily routines, prolonged isolation, and school closures deteriorated the psychological well-being of young people, with more severe effects in historically marginalized communities (1). These factors, along with the disruption of emotional and caregiving networks, contributed to an increase in symptoms such as stress, anxiety, and sadness (2–4), and highlighted the fragility of social protection systems for adolescents impacted by gender, class, and ethnicity (5–7).

Although the concepts of mental health and emotional health are often used interchangeably (8), this study draws a key distinction. The World Health Organization (WHO) defines mental health as a state of optimal individual functioning that allows one to work productively and adapt to the social environment (9). This definition has been widely criticized for reproducing individualistic and productivity logics inherent to the capitalist system (10, 11). Likewise, the dominant notion of mental health is anchored in the biomedical model, which focuses on individual diagnosis, the medicalization of suffering, and the functional restoration of the subject to the productive order (12). This vision, centered on the subject's functionality for the system, is why we align with the critique from the *Children's Understandings of Well-Being* (CUWb) research, which seeks to shift the focus from *well-becoming* (what the young person will become) to *well-being* (the adolescent's experience in the present) (13).

This model has been challenged for its reductionist and decontextualized nature, which tends to stigmatize emotional states considered abnormal or unproductive (14, 15), and to obscure the social determinants of distress (16, 17). For this reason, our study adopts the concept of *emotional health* from the paradigm of *collective health* (18), as a way of understanding well-being beyond the individual, most used in Latin America. Rather than being reduced to an internal balance or the achievement of productivity, emotional health is understood as a relational and situated process, shaped by material living conditions, affective ties, the social environment, and structures of inequality. Furthermore, this study aligns with the critical perspective of CUWb, viewing emotional health as an open concept that must be delimited by the empirical evidence provided by the young people themselves (13). This perspective allows suffering and well-being to be approached as phenomena that express not only personal histories but also structural tensions. Thus, the conception of emotional health offers a more holistic and interrelational view that integrates individual, social, cultural, and structural aspects of well-being. Well-being, in this framework, is considered a *bottom-up* effect (ascending), which means it emerges from concrete living conditions and manifests as a shared and stable response to common environmental and relational conditions (19). Recognizing this relational and contextual dimension is crucial to address adolescents as *expert subjects* in their own lives (20). It also involves self-awareness and the management of one's own emotionality (21, 22).

This perspective emphasizes the ability to recognize and express emotions without confining them to diagnostic categories or value judgments, integrating both personal and collective subjectivity. Emotional health opens a space for dialogue with adolescents that challenges the stigmatization of everyday distress and proposes approaches based on recognition, attentive listening, and the narration of personal experience (11). From this perspective, intersectionality is also incorporated as an analytical tool to understand how multiple systems of oppression (patriarchy, racism, neoliberalism) differently shape the emotional trajectories of adolescent girls (23–25). Recognizing these intersections shifts the focus away from individual pathology toward the structural conditions that enable or constrain well-being.

We have chosen to use an intersectional framework rather than the social determinants of health perspective, as we believe the former offers a richer and more embodied approach, allowing us to understand how structures of inequality are assembled and interrelated in the life trajectories of young women. Likewise, this perspective draws on debates from black and decolonial feminisms, consistent with the study setting, and triggers the need to incorporate the role of structural violence and oppression into the analysis of young women's emotional health (26). This perspective contributes to feminist debates that value the role of emotions and affections in people's health and daily lives (27).

In accordance with this framework, the need arises to conduct this study through *participatory action research*, aiming to challenge the power dynamics between researchers and participants in order to generate processes of awareness and social transformation (28). Specifically, the use of photovoice is proposed as a methodological tool that explores visual languages through which adolescent girls express and represent emotions that may be difficult to verbalize. Photography is thus understood as an expressive and agentic practice that enables the creation of personal narratives often silenced by more hierarchical or adult-centered methodologies. This method aligns with the critical imperative of well-being research, which positions adolescents as social actors whose subjective experiences and narratives provide knowledge of how adolescence is socially constructed (13). Indeed, previous research has shown that imagery can articulate affective memories, social critique, and collective aspirations, especially among young people historically excluded from knowledge production processes (29, 30). In the field of public health, photovoice has emerged as a meaningful tool for fostering open and honest conversations and strengthening connections between different groups (30). In this way, photovoice is conceived not only as a data collection technique, but as a political and epistemological act that recognizes the legitimacy of other ways of feeling and knowing.

Despite the growing interest in the psycho-emotional effects of the syndemic, a significant gap remains in studies that address well-being from the voices of adolescents themselves, especially in contexts of high cultural diversity, structural poverty, migration, and gender inequality (11). This gap underscores the need to address adolescents as *expert subjects* in their own lives (20). Therefore, this study set out to explore how a group of adolescents attending a girls‘ school located in an area characterized by inequality and cultural diversity experience and make sense of their emotional well-being in everyday life. Through a participatory and visual methodology, the study aimed to understand how these experiences relate to their relationships, their contexts, and the ways they express emotions through imagery. Rather than identifying risk factors or diagnoses, this work focuses on recognizing agency, care strategies, and unique ways of constructing emotional meaning from adolescents' perspectives. Listening to, acknowledging, and incorporating their voices is both an ethical and political condition for building fairer collective responses in health.

## Methodology

The study employed the Photovoice method as a strategy to explore adolescents' emotional health from their own perspective. It was implemented not only as a data collection technique, but as an epistemological tool aimed at challenging adult-centrism, legitimizing subaltern knowledge, and promoting youth agency in knowledge production.

### Context

Chile, considered a high-income country according to the World Bank (31), is also characterized as one of the most unequal countries in the OECD, with a Gini index of 0.43 in 2022 (32). Although the country has shown a trend of progressive reduction in income and multidimensional poverty, this has had the opposite effect in immigrant households, where one in three people born outside Chile live in multidimensional poverty, almost double the rate for Chilean nationals. Food insecurity in households headed by someone born outside Chile reaches 28.6%, 10 percentage points higher than in households headed by Chilean nationals (33).

Meanwhile, 20.8% of children and adolescents under the age of 14 who were born outside Chile do not have health insurance, compared to 2.6% of their peers born in Chile (34). In turn, 77.8% of young people between the ages of 15 and 19 are studying, compared to 57% of their foreign contemporaries (35).

In Chile, the COVID-19 health crisis overlapped with the 2019 social uprising, which challenged the neoliberal model and the forms of precarity shaping everyday life (36). The education system, already under strain, faced an extended closure of nearly 2 years. This led to an unequal transition to virtual and hybrid learning formats, with a full return to in-person classes only occurring in March 2022—without clear protocols to mitigate the psychosocial impact on the educational community (37). The research was conducted in a public, single-sex secondary school located in the municipality of Recoleta, Santiago de Chile, during December 2022. The school is situated in an area characterized by ethno-cultural diversity and high socioeconomic vulnerability (38). Sixteen percent of residents are international migrants, and 11% belong to indigenous peoples (38). The research team carried out three in-person sessions, each lasting approximately 3 h and spaced 1 week apart. All sessions were video recorded, with prior consent from participants and their legal guardians.

### Photovoice

Photovoice is a participatory action research method aimed at transforming people's realities through the use of photography and storytelling as a means of promoting personal awareness and collective dialogue around shared experiences (29). In other words, Photovoice uses visual material (photographs) as a platform for reflection and a channel for self-expression. This method has been shown to foster community engagement and empowerment, generating social impact—from the development of critical awareness to the promotion of collective action.

One of Photovoice's defining features is its ability to access everyday contexts that are often inaccessible, and to perceive social issues from the participants' point of view. In addition, the method's flexibility makes it adaptable to different research objectives, groups, and communities, allowing for goals to be redefined during fieldwork. Its acceptability is another strength, particularly given its capacity to motivate participants, especially youth, through its participatory approach and the use of cameras (29, 30). A common form of collective action derived from Photovoice is the organization of photographic exhibitions to reach a broader public. Among youth, it is also common to create posters featuring the captured images as a means of protest or to write letters addressed to key stakeholders (30). Overall, the method seeks to raise awareness and disseminate findings to various sectors of society.

### Participants

Eleven first-year secondary school adolescents participated in the study (nine identified as cisgender girls and two as non-binary individuals), aged between 14 and 15 years. They were selected through intentional sampling, based on their commitment to participate in all phases of the project. The participants came from diverse family backgrounds and lived in different municipalities of Santiago (Table 1). This diversity was considered a key element for highlighting the intersections of migration, social class, gender, and ethnicity in the shaping of emotional well-being. In this line, the study prioritizes the credibility and depth of the participatory process over sample size, aiming for a situated understanding of the phenomenon rather than generalization of the results (39). This approach allows for the transferability of findings through the contextualization of participants' experiences, enabling reflective dialogue with other communities that share similar structural characteristics.

**Table 1:** Photovoice participants by pseudonym, age, municipality of residence, and family country of origin.

**Pseudonym**	**Age (years)**	**Municipality**	**Family country of origin**
Niig	14	Recoleta	Peruvian
YR	15	Independencia	Ecuadorian
Belu	14	Independencia	Chilean
Nuri	14	Maipú	Chilean
Virgo	14	Independencia	Chilean
Massinnie	14	Independencia	Peruvian
Aiko	14	Recoleta	Chilean
Jhalieta	14	Recoleta	Bolivian
Nazareth	15	La Cisterna	Venezuelan
Yén	15	P. Aguirre Cerda	Chilean
Nana	15	Conchalí	Chilean

Compiled by the authors based on data provided by the participants.

### Data collection and analysis process

The data collection process was organized into three phases. The first session consisted of a collective dialogue to reflect on the concept of emotional health and methods of emotional regulation based on personal experiences. A workshop led by a social photographer was also held, offering basic photography techniques and tools, with a focus on ethical recording practices. At the end of the session, participants were asked to take or gather photographs of places, actions, people, or situations that contributed to their emotional well-being.

In the second and third sessions, the 24 photographs were collectively analyzed. Each participant presented between one and six images, describing the context and emotions associated with them, which facilitated peer discussion. Following this preliminary phase, the research team conducted a deep manual collaborative coding process. Adhering to the coding paradigm (40), the analysis progressed from open coding—identifying broad categories from the photographs and narratives—to axial coding, where specific subcategories were refined based on their properties and dimensions. Disagreements regarding coding were resolved through dialogic consensus meetings among the researchers, serving as a strategy to prevent analytical drift and ensure interpretive consistency (investigator triangulation). Finally, the emerging thematic areas were validated in a participatory group session where adolescents grouped the concepts, which were then deepened and formalized by the researchers into the final analytical categories.

This approach aims to challenge traditional research hierarchies by positioning adolescents as analysts of their shared experiences and recognizing visual expression as a legitimate form of knowledge. The combination of narratives, photographs, and peer discussions helped to enrich the conversation on emotional health, shifting the focus from the individual to the collective level.

### Reflexivity and analytical positionality

Given the political nature of this study, the research team adopted an explicit analytical position grounded in Collective Health and Intersectional Feminism. We engaged in the research with a theoretical commitment to questioning the pathologization and individualization of adolescent distress, viewing emotional health instead through structural, collective, and situated lenses. This stance implies recognizing that our analysis is not neutral but seeks to validate affective dimensions often marginalized by hegemonic biomedical models.

To ensure methodological rigor, we adhered to the principle of epistemological vigilance (41). This approach demands a continuous questioning of the relationship between the researcher and the object of study, ensuring that techniques are not applied uncritically. We exercised this vigilance to identify and rupture pre-existing academic or adult-centric assumptions that might obscure the adolescents' meanings. Consequently, our methodological strategies—such as the dialogic consensus process and constant team reflexivity—functioned not merely as procedural steps, but as active mechanisms of epistemological control. These spaces allowed us to construct analytical categories derived directly from the participants' empirical reality, rather than imposing external theoretical frameworks upon them.

## Results

The analysis of the images and narratives created by the participants gave rise to seven thematic categories representing different dimensions of emotional well-being. These categories, constructed collectively, reflect both pleasurable and distressing experiences, allowing for the identification of tensions that affect adolescents in contexts of inequality. Each thematic axis combines stories, emotions, and symbolism that, beyond individual perspectives, provide insight into socio-emotional processes from a situated standpoint.

The articulation of these findings positions emotional well-being from the Collective Health paradigm, understanding it as a fundamentally relational and situated construct that is built in contexts of precariousness and structural vulnerability (18). The results, by reflecting the experience of adolescents in a context marked by migration and cultural diversity, demonstrate how structural inequalities (the feminization of care, precarious work, or adult-centrism) strain the intimate and collective spheres. Within this framework, the categories converge to highlight adolescent agency, revealing how young women forge systems of care and resistance—whether with peers, animals, or through political action—as a response to the insufficiency of institutional support.

Crucially, the diversity of the thematic axes (from Desolation to Collective Demands) challenges the individualistic and pathologizing vision that tends to medicalize distress, legitimizing unpleasant emotions as an integral part of emotional development. In this way, the analysis of the seven dimensions allows emotional health to be understood not as a state of internal balance, but as a dynamic and political process that demands recognition, listening, and social justice.

### The value of friendship

Friendship was identified as a key dimension of emotional well-being among the participants. The images and narratives illustrated how connection and coexistence among peers serve as sources of emotional support, mutual recognition, and the building of trust—especially in a life stage marked by transitions, such as adolescence, and the rebuilding of social bonds after the pandemic.

**Figure 1:**
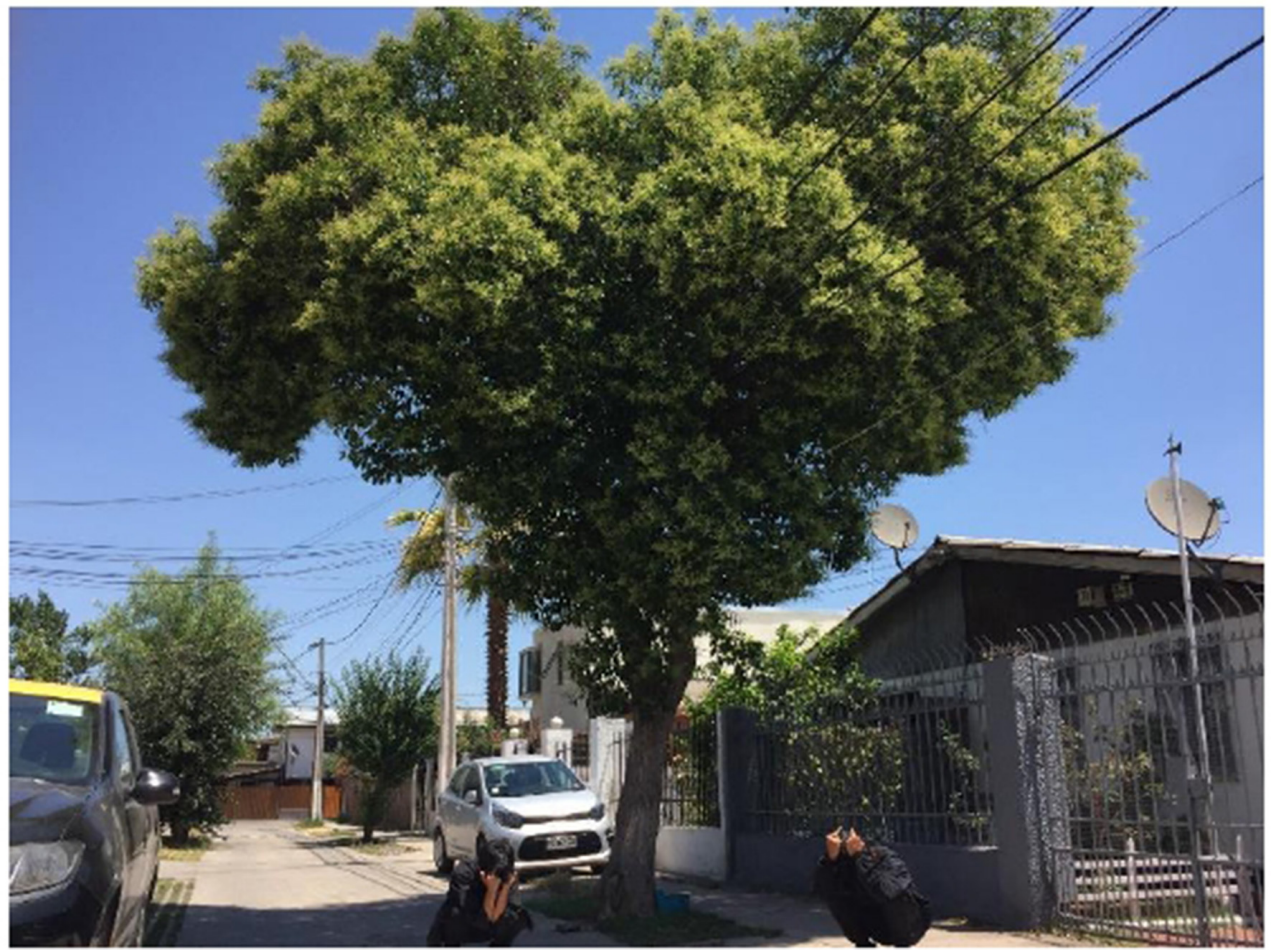
Friendship in a new heart. Source: Photograph and narrative by Virgo. “The photo represents a moment of friendship, perhaps a new beginning, happiness. It symbolizes joy, companionship, connection, freedom, affection, heart, a moment of peace, laughter, good memories, energy, love.”

From the adolescents' perspective, friendship was not only a source of fun, relaxation, and shared adventures (Figure 1), but also an emotional refuge where they could express vulnerability without fear of judgment from adults or family members. Peer relationships were described as safe and egalitarian, in contrast to the often-hierarchical dynamics of family and school settings.

In a context marked by high mobility and perceptions of neighborhood insecurity, friendships played a central role in fostering a sense of belonging and emotional safety. Although school was acknowledged as a space that encouraged peer interaction, the emphasis was placed on how adolescents valued friendship as an independent source of emotional well-being—where they felt heard, supported, and accompanied in their personal processes, extending beyond the school environment.

From a critical perspective, these findings call into question how emotional well-being in adolescence is sustained by affective networks that are not always recognized by institutional frameworks in health or education. They also highlight the capacity of young people to build spaces of mutual care in the absence of adult support, emphasizing the need for perspectives that legitimize and integrate these relational forms of care and support, which are positioned as a fundamental support system in the face of the affective distance observed in the family sphere.

### Collective demands

**Figure 2:**
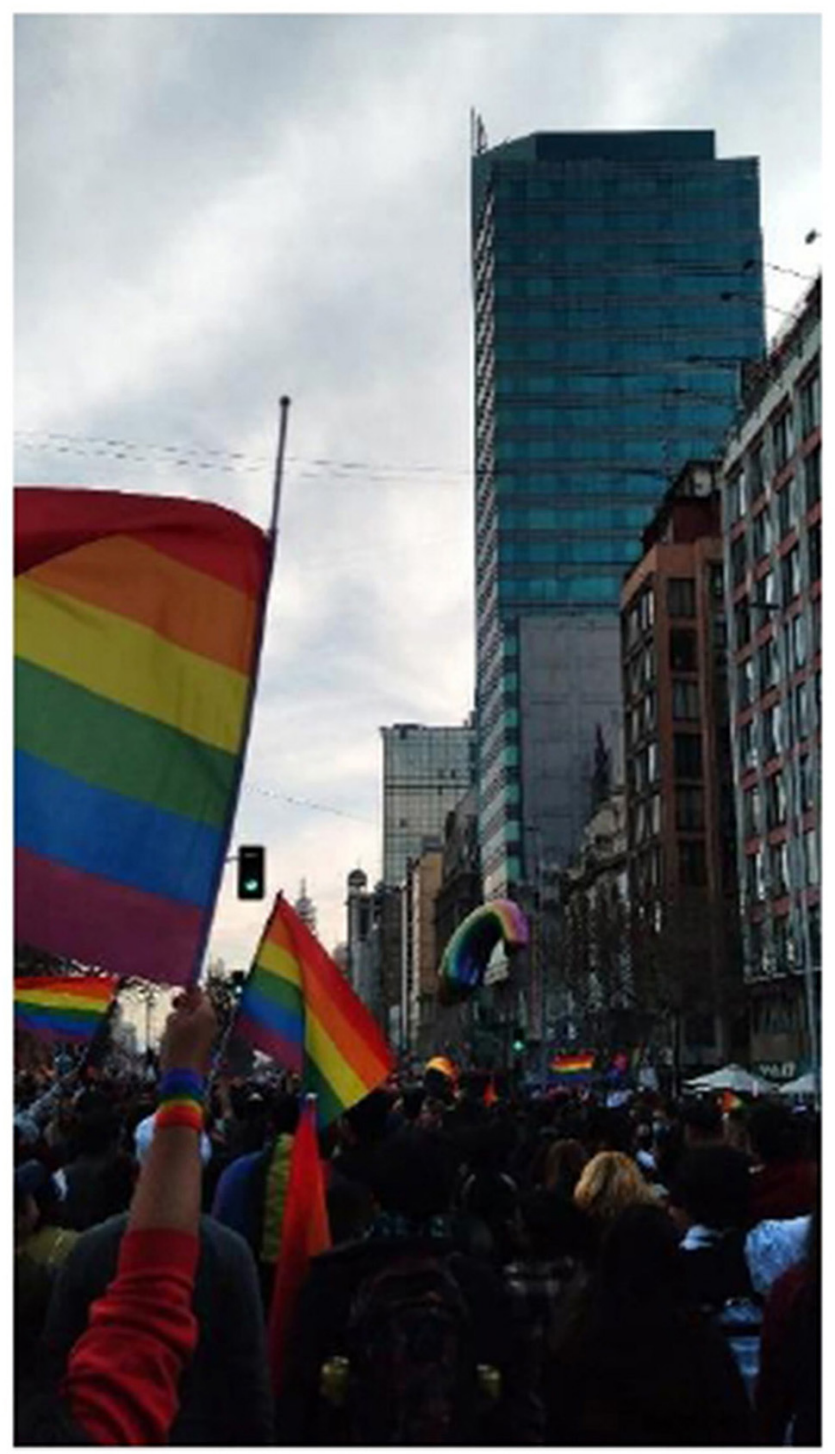
My freedom without judgment. Source: Photograph and narrative by Nuri. “I took this photo because that day was and still is very important in my life. It was full of joy, and I felt free. I was in a place where I felt safe and not judged.”

The photographs and narratives in this section on collective demands are linked to the adolescents' participation in marches and social demonstrations, particularly the LGBTQIA+ Pride March and student protests. In both experiences, participants described feelings of freedom, empowerment, and safety in being part of spaces where their identities, voices, and struggles were collectively recognized (Figure 2).

Emotional well-being emerged in connection with political action and a sense of belonging to a collective that challenges conservative social norms responsible for exclusion. The emotions tied to these activities encompassed not only celebration and liberation, but also a sense of relief from the stigma, discrimination, and silence experienced in everyday life.

Marches and demonstrations served as spaces of subjective validation, where the adolescents encountered others with similar identities, strengthened their self-esteem, and reinterpreted prior experiences of exclusion and lack of recognition. Capturing these moments in photographs and selecting them as significant to their emotional well-being indicates not only their personal importance but also a way of expressing resistance and agency through visual representation.

From a structural perspective, these findings underscore the need to understand emotional health not just as an internal state, but as a political construction that values recognition, justice, and the ability to act upon one's social environment. In contexts where adolescents do not feel represented or protected by traditional institutions, activism emerges as a legitimate and transformative way of caring, both individually and collectively, channeling the distress derived from exclusion and social stigma toward empowerment, in contrast to the experiences of anguish and emptiness lived in the individual sphere.

### Four-legged affection

**Figure 3:**
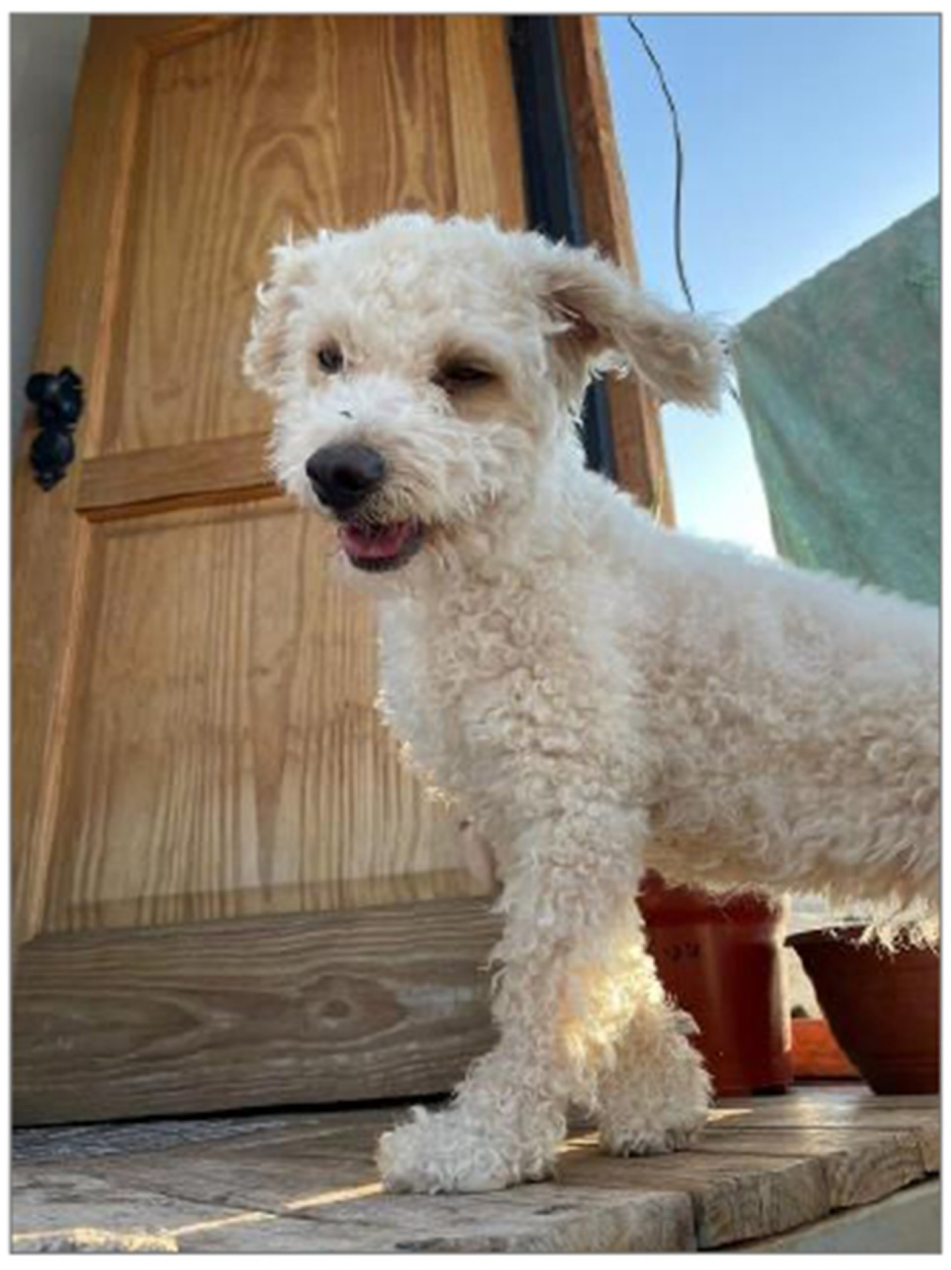
Love, peace, and tranquility. Source: Photograph and narrative by Belu. “This photo is of my little dog Linda. I took it to have a memory of her since I know dogs don't live forever. Also, in the photo you can see how the sunlight touches her, and she looks very beautiful.”

Relationships with companion animals emerged as a significant source of emotional well-being for the adolescents. The photographs showed everyday interactions with dogs and cats, and the accompanying narratives expressed feelings of joy, calm, unconditional companionship, and emotional comfort (Figure 3).

During a life stage marked by insecurity, academic pressure, and conflicts with adult figures, companion animals appeared as affective beings offering a bond free of judgment or demands. For some adolescents, these relationships were described as true friendships or even as their primary source of emotional support during times of stress, tension, or loneliness.

Physical contact, play, and even simply observing their dogs and cats provided sensations of peace and distraction. A distinct emotional tone emerged when talking about their pets, revealing an affective need to maintain that bond. Anticipatory grief also surfaced, as some adolescents expressed sadness at the thought that animals have shorter lifespans than humans, intensifying the value they place on that emotional connection.

This finding expands the understanding of affective bonds in adolescence beyond human relationships, encouraging reflection on the role of animals as part of everyday care networks. From a collective health perspective, recognizing animals as emotional support systems highlights non-conventional forms of well-being that challenge the biomedical paradigm, which is primarily centered on professional intervention, and aligns with the search for non-hierarchical and unconditional bonds that characterize other forms of adolescent emotional support.

### Treasure family memories

**Figure 4:**
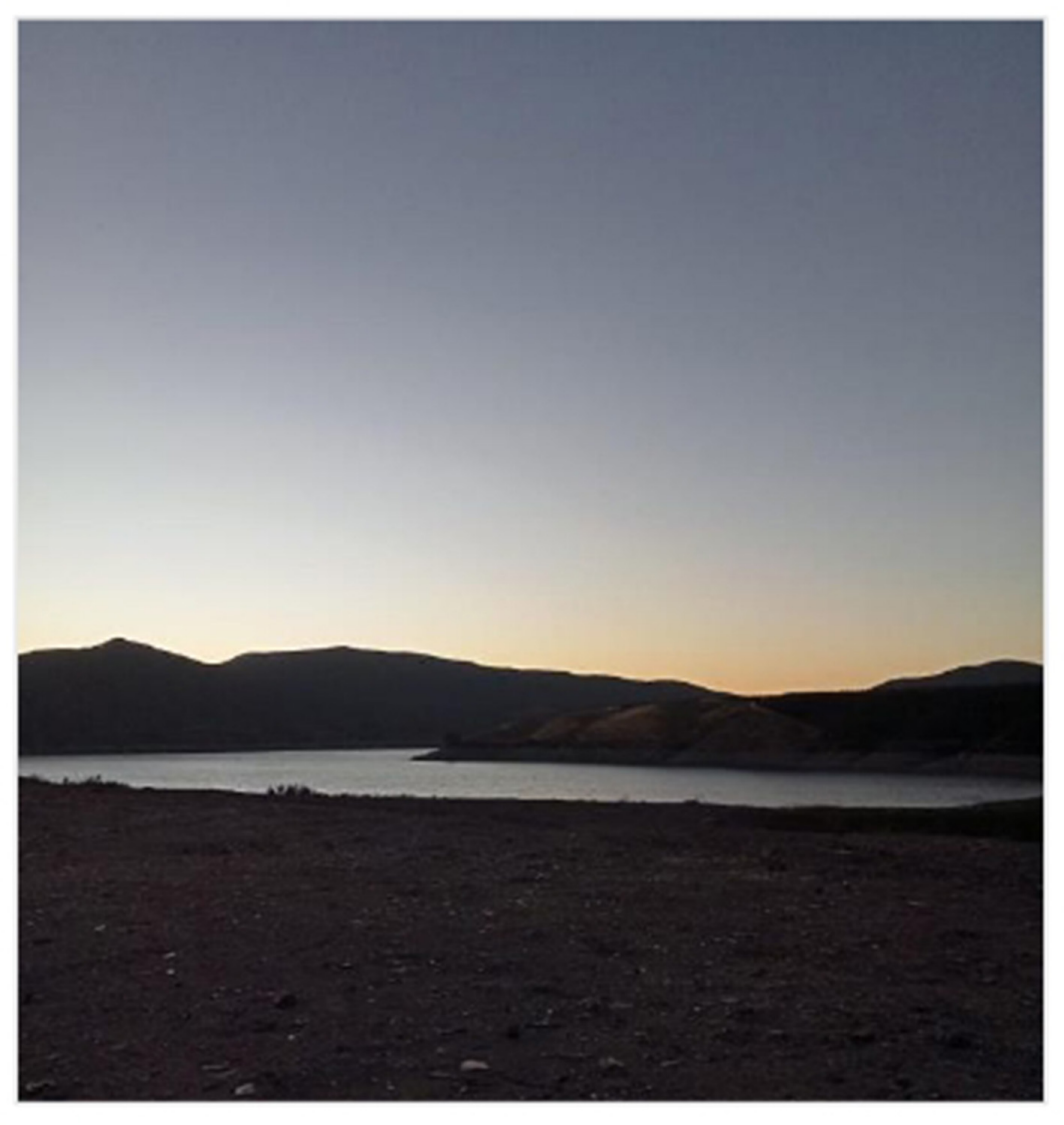
Family moments. Source: Photograph and narrative by YR. “This image reminds me of an unforgettable outing I shared with people I love, and every time I see it, I feel like I travel back to that incredible moment I experienced.”

The images associated with this category evoke shared family memories during special moments such as outings, celebrations, or trips (Figure 4). These memories were recalled with affection and nostalgia, highlighting emotional bonding in contexts where adult time and emotional availability are often limited.

Adolescents highlighted close relationships with older relatives, such as grandmothers, and with younger siblings, while maternal and paternal figures were not always mentioned as active sources of emotional support. Several narratives emphasized that the labor and domestic responsibilities of parents often hindered the development of close, day-to-day connections. This emotional distance shaped the nature of adolescents' relationships with their parents, who, due to their absence, were primarily perceived through a disciplinary lens. As a result, interactions tended to focus on academic performance or household duties, reinforcing their role as daughters and often relegating their emotional needs of the young women. These findings suggest that shared family moments are especially valued precisely because they are rare and contrast with daily experiences in which parental bonds are marked more by obligation than by emotional closeness.

Additionally, some participants from migrant families expressed a strong desire to reunite with relatives who remained in their countries of origin. The physical distance made them feel there was an unresolved bond, as if it were a pause until they could meet again. These accounts revealed how emotional well-being is also affected by physical separation from loved ones, especially when reunion depends on circumstances beyond their control.

This category highlights how structural conditions—such as job insecurity, migration, and the feminization of care—shape the ways in which emotional well-being is constructed and sustained within the family sphere. While family memories evoke affection and a sense of refuge, they also reveal their intermittent nature, strained by the pressures of time, labor, and absence—particularly affecting the most vulnerable social sectors, generating an emotional distance that drives adolescents to actively seek support and security in their peer networks and other collective spaces.

### The school as a safe place

The images associated with this category depicted the school as a space where adolescents felt calm, protected, and valued. Despite the uncertainty surrounding the return to in-person classes, many participants described the school environment as a welcoming place—one that fostered new friendships and enabled them to connect with peers in an atmosphere of respect and diversity.

**Figure 5:**
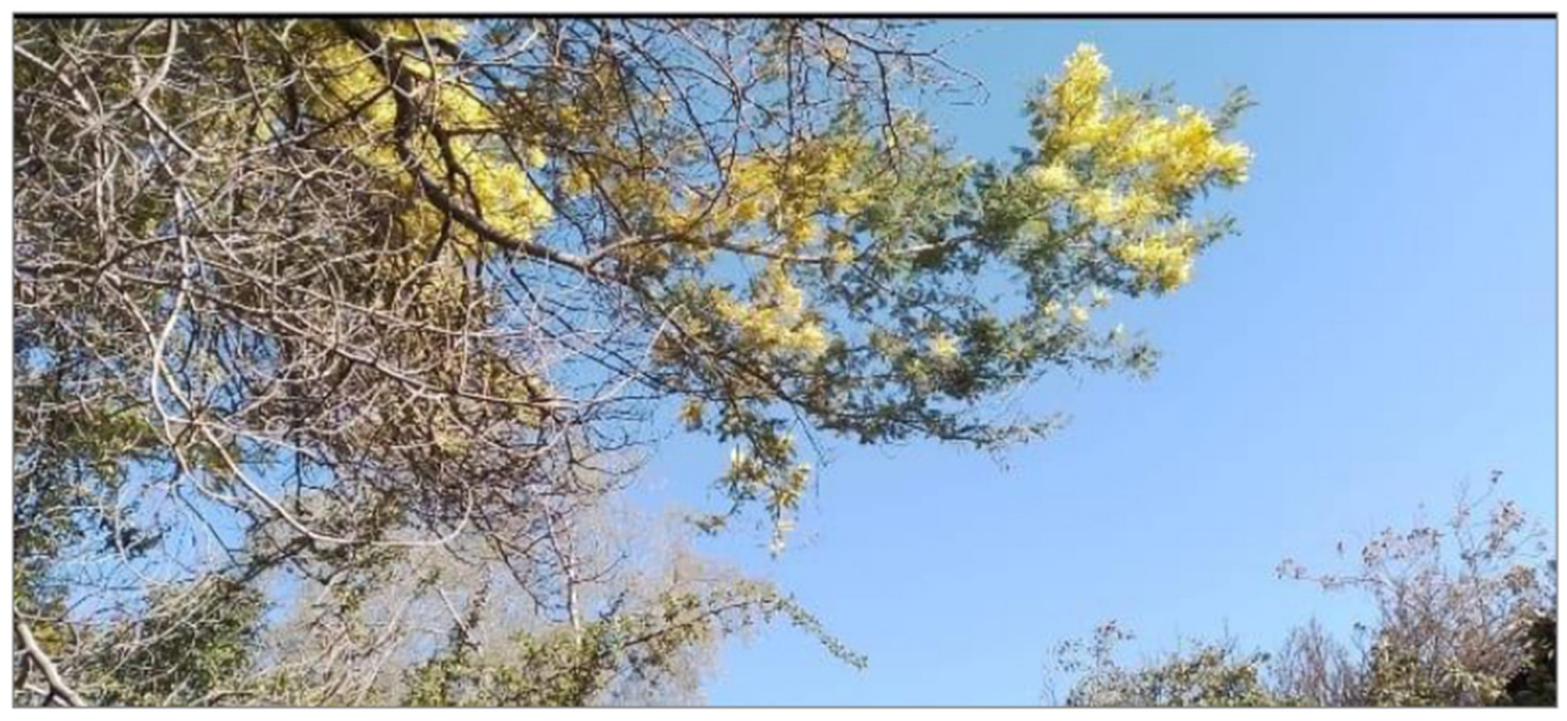
Comfortable place. Source: Photograph and narrative by Niig. “For me, this photo represents peace, calm, and harmony since it was taken in a place that made me feel that way—the playground of my school. What made me feel this way is more the school than the playground itself, but I couldn't find a more perfect place to represent how I felt when I arrived at school.”

The fact that the institution was single sex was particularly valued, as it fostered an environment perceived as less hostile and free from the violence or harassment often experienced in coeducational schools. Participants also highlighted material features such as green spaces and recreational areas (Figure 5), along with the respectful and supportive attitude of the teaching staff, all of which contributed to a sense of safety and well-being. Among the students of migrant origin, the school was described as a space that facilitated meeting and connecting with other students from the same country, which strengthened the sense of belonging and reduced feelings of isolation or shame due to discrimination. Additionally, school festivities that recognized students' cultural diversity were highly valued, as they helped reinforce their identities and highlight the richness of the intercultural environment.

This category prompts reflection on the role the school can play in contexts of social vulnerability beyond its educational function. For many adolescents, school represented a space that offered tranquility and security. The existence of green areas and recreational spaces was especially appreciated, as they were seen as places for gathering, resting, and emotional well-being. However, access to safe and emotionally supportive school spaces is not equitably guaranteed within the Chilean educational system. Adequate infrastructure and the recognition of cultural diversity are not systemic features of the system but rather outcomes that depend on the vision and commitment of specific school communities. This situation reveals an institutional fragility that reproduces inequalities, underlining that institutional safety is based on the school's capacity to compensate for the system's deficiencies, actively facilitating peer bonds and identity recognition that the social structure denies.

### Nature: a reflection of emotions

**Figure 6:**
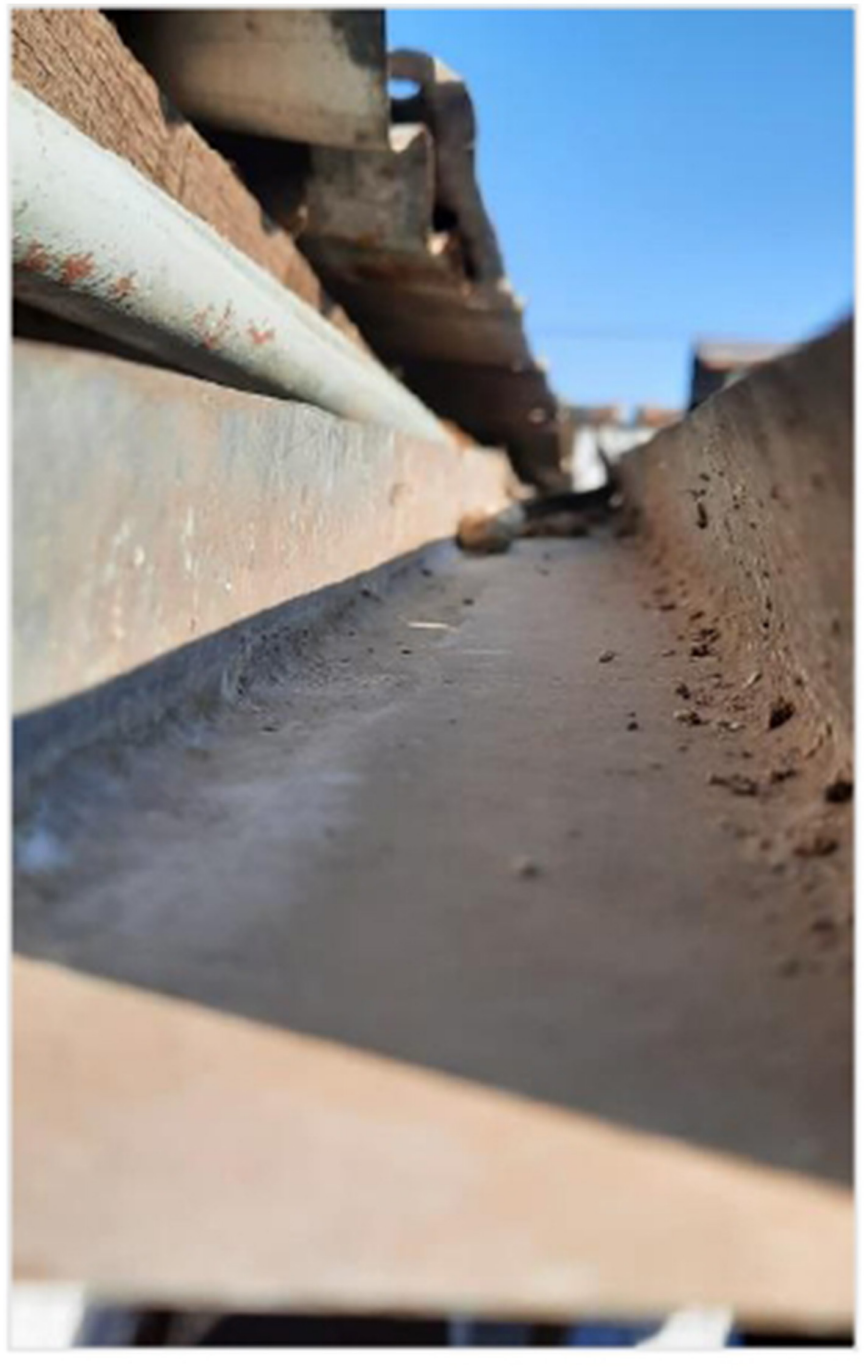
Waiting for the rain. Source: Photograph and narrative by Jhalieta. “This photo was taken at my home; it represents water scarcity and global warming. That's why it shows a gutter on a hot and dry day in Chile, because basically without rain the gutter does not fulfill its function.”

The images related to nature were used by the adolescents to express complex emotions, both pleasant and distressing. Nature was symbolically portrayed as a space of refuge, freedom, and connection associated with trips or experiences beyond everyday life, but also as a source of concern and uncertainty, mainly regarding environmental degradation (Figure 6).

Some photographs showed landscapes such as the sea, trees, or drought-affected areas, which were described as representations of emotional states. One participant, for instance, compared the waves of the sea to sudden emotional shifts that lacked an identifiable cause. This analogy between the natural world and internal experience enabled the adolescents to explore aspects of their emotional lives that were sometimes difficult to explain with words alone. Alongside this symbolic use, concrete concerns related to the climate crisis also emerged. Several adolescents expressed anxiety about environmental destruction and the planet's future. This ecological dimension of distress shows how emotional well-being is not isolated from global contexts, and how adolescents in the Global South are sensitive to structural processes that affect their present and future.

From a critical perspective, this category broadens the analysis of emotional well-being by including the relationship with the natural environment as a significant element. Nature not only serves as a stage for emotional projection but also as an indicator of ecological consciousness that links the person with the collective. Recognizing this connection is crucial for informing public policies that integrate environmental care into emotional health strategies for adolescents, functioning as a space for emotional release and symbolization that, like non-human bonds, offers a judgment-free refuge for the expression of complex, global, and inarticulate emotions.

### Desolation

Although the initial goal of this study was to identify spaces and relationships that contributed to the participants' emotional well-being, the images and narratives also revealed experiences expressing sadness, emptiness, or anguish. This category brings together unpleasant experiences that, far from being marginal, were recognized by the adolescents as integral to their process of personal growth and internal reflection.

**Figure 7:**
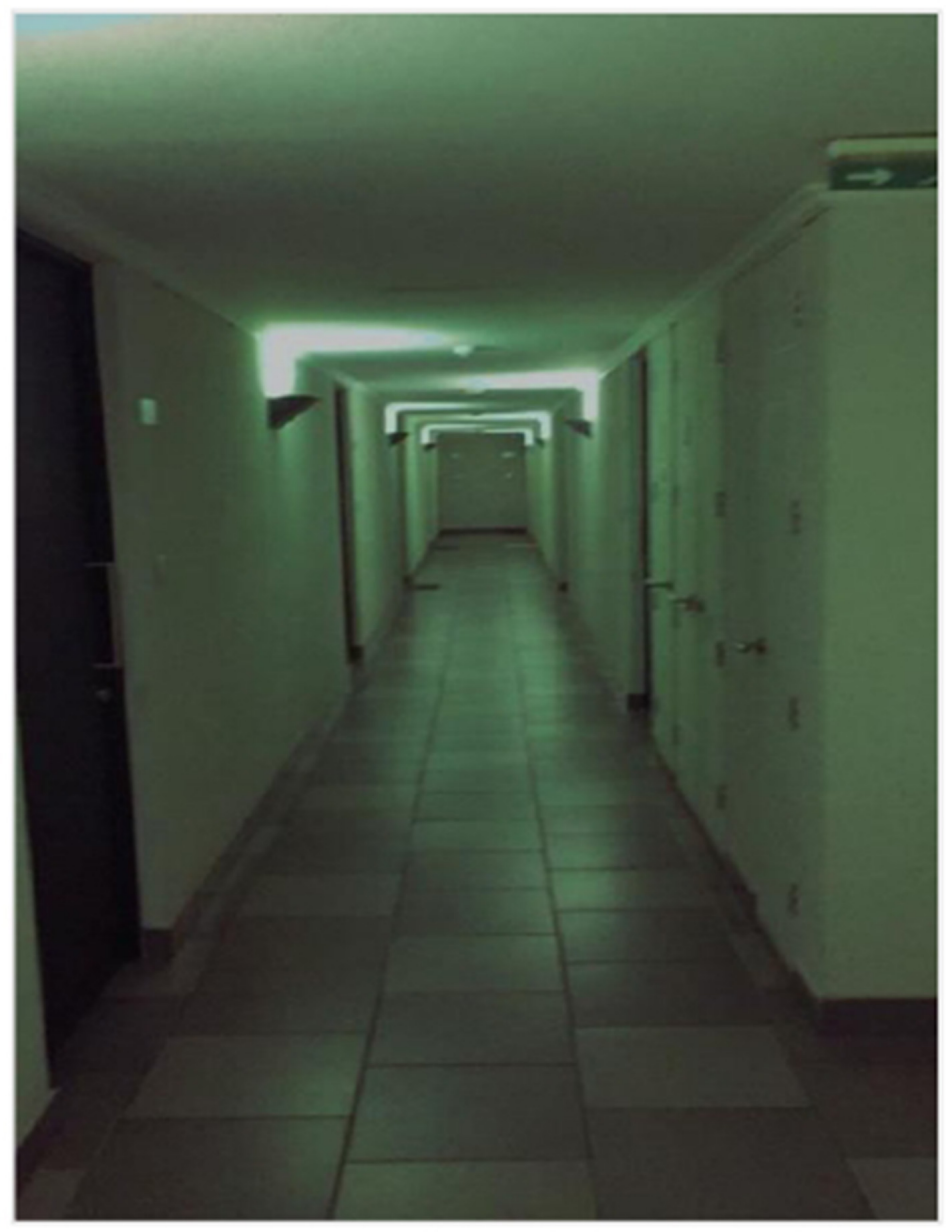
No return. Source: Photograph and narrative by Virgo. The fear of facing your darkest side but continuing to move forward to a better place.

The photographs portrayed solitary spaces (Figure 7), broken or discarded objects, or precarious places, accompanied by stories of emotional ruptures, economic hardship, uncertainty, and nostalgia.

Without pathologizing these states, the adolescents interpreted them as necessary moments to better understand themselves, make decisions, or reframe painful experiences. This interpretation contrasts with adult-centered views that tend to medicalize adolescent distress and contributes to a more complex understanding of emotional health as a dynamic process that includes both joy and pain.

This category challenges discourses that associate emotional health solely with visible or positive well-being. Desolation appears here as a legitimate way of experiencing the world, requiring listening, accompaniment, and validation. Acknowledging these emotions without stigmatizing them opens the possibility of addressing them from a collective and caring perspective, recognizing adolescent suffering as part of their emotional growth, and positioning emptiness and anguish as the intimate manifestation of the structural tensions that the other categories actively seek to mitigate through mutual support and political agency.

## Dissemination and feedback

Beyond the collective knowledge-building process, this research incorporated a strategy to give back to the participants and their educational community. Two dissemination products were developed for school, health, educational, and academic audiences: a documentary video of the photovoice process (https://www.youtube.com/watch?v=w9kAeNwRK5o) created with the support of a social photographer and the adolescents themselves; and a photo book (https://libros.uchile.cl/1547) compiling all selected images, accompanying narratives, and the collective thematic analysis.

Both materials were shared through institutional networks (Open University of Recoleta, Faculty of Social Sciences at the University of Chile, and IDIAPJGol in Spain) and used as educational and community resources. One year after the fieldwork (December 2023), a feedback meeting was held at the high school with the adolescent participants and their classmates, where the video and photo book were publicly presented. Most participants attended, except for two who were no longer enrolled at the institution.

During this event, a meaningful dialogue emerged: memories, expressions of gratitude, and proposals for future activities were shared, including the desire to incorporate bodywork as part of the emotional approach. This gathering not only reinforced ties with the community but reaffirmed the importance of returning results to those who shared their experiences as an essential part of the photovoice methodology. Sharing results is not a symbolic closure but a vital stage in the participatory process, where knowledge is returned to those who generated it, fostering recognition, ownership, and the continuation of collective dialogue.

## Discussion

The adolescents in this study shared deep and thoughtful reflections on what they consider emotional health to be and the factors that contribute to their well-being through visual imagery and collective reflection. They highlighted meaningful moments and the importance of relationships, as well as more painful experiences that are nonetheless part of the continuum of emotional well-being. The use of photographs taken by the participants themselves fostered self-expression, individual and group responsibility regarding the issues that resonated with them, as well as creativity and attentive listening. This strategy for generating collective knowledge proved particularly effective, as it encouraged dialogue not only on specific aspects, but also on the interpretation of the photographs and their meanings. Participants pointed out the lack of spaces for active listening with minimal intervention from adults.

Key elements identified as essential for emotional well-being included connections with others, relationships with companion animals, the role of the natural environment, and a sense of safety in their new educational institution. Additionally, desolation emerged as an emotion which, although perceived as unpleasant, is necessary and part of experience and vital growth.

According to this photovoice study, unusual events were particularly valued. The opportunity to enjoy leisure time with family was highlighted, precious moments captured in photographs showing places or activities shared with them. This centrality of family and quality time aligns with qualitative evidence that support, understanding, and family bonds are fundamental to the well-being of young people (19). Furthermore, most participating adolescents acknowledged that their families care about their well-being and school performance. However, they also expressed themselves not feeling fully heard by their families, perceiving limited recognition of their emotions and personal experiences. Many expressed a desire to be more acknowledged and validated, particularly by their mothers and fathers. This factor is also related to the time and availability families have to create such spaces, both in daily life and when undertaking new activities, often limited by work- related obligations. This reflects the tension between care and precariousness, as the demands of economically supporting the family limit the time and probably the quality of time available to accompany adolescents. Thus, life under a neoliberal model is marked by vulnerability, creating dependency and reinforcing the need for others to help make life more livable. As such, relationships of care and mutual responsibility become central (42). These results allow us to assess how, from an intersectional perspective, different specific experiences intersect, reflecting overlapping systems of oppression. Young people from the same background demonstrate that they possess multiple identities that intersect to generate unique experiences and inequities in emotional health (43).

Second, the relationship with companion animals such as dogs and cats with whom adolescents live daily gained special importance, emerging as a particularly relevant factor for everyday emotional well-being. Their role as affective anchors within intimate spaces was emphasized, as they were associated with unconditional friendship, companionship, entertainment, and a sense of calm. The relevance of these non-human affective bonds reinforces the bottom-up vision of well-being, where satisfaction with specific life domains and concrete relational conditions are the basis for general emotional health (19). In some cases, companion animals were described as best friends, valued precisely because they do not express social judgments. This contrasts with human relationships, whether with peers or adults. Recent studies highlight the significance of the human–animal bond during periods of crisis, showing that emotional support and companionship from animals can help reduce stress and feelings of loneliness (44–46). Other research has underscored the role of animals during lockdowns, particularly for individuals living alone or with minors (47). In relation to adolescents experiencing anxious or melancholic emotions, Salazar (48) affirms the positive impact of companion animals on emotional health, considering this bond a therapeutic tool that fosters emotional balance through love, trust, play, companionship, and mutual support, especially in contexts of loneliness or stress.

Along the same lines, nature and landscapes emerged as sources of expression and tranquility. This finding resonates with research that highlights the relevance of spatiality and living environments in shaping subjective well-being (49). Through symbolically rich images, adolescents reflected on social and environmental issues that caused them concern. In this context, the environment held a dual meaning representing both desire and worry. Research in low socioeconomic status communities similar to ours emphasized that the presence of natural spaces is crucial for well-being, offering a refuge from urban environments perceived as hostile (50). Their intense valuing of green areas or patios as essential for well-being has also been documented in Chilean literature as a marker of inequality (20). For instance, the phenomenon of eco-anxiety, linked to the climate crisis and the current development model, is especially common among young people and communities in the Global South (51). The strong emotional connections with animals and the concern for environmental degradation emphasize the need to recognize the role of ecology in the emotional health of adolescents. This perspective challenges the anthropocentric focus of mainstream health models and highlights the importance of caring for the environment as an essential part of both personal and collective well-being. Human health, the well-being of other species, and planetary health are deeply interconnected (47, 52).

Third, the importance of belonging to an environment that generates safety, such as the school institution from a female and non-binary perspective, was recognized for emotional well-being. The school was described as a safe public space that promotes healthy, harmonious relationships and meaningful forms of connection. This valuing is supported by global findings that identify friendship as the most valued and continuous well-being experience across all adolescent age groups, while school is simultaneously a source of socialization and tension (19). The role of teachers as mediators in communication and in managing tensions within the community was highlighted. However, literature also indicates that adolescents from low socioeconomic status contexts often report greater discontinuity and poorer relationships with school adults, reinforcing their demand to be heard (20). Participants linked many of the school's positive qualities to the fact that it was an all-girls educational setting. This view was justified by participants' previous experiences. It was mentioned that in other schools, violence occurred, mainly by male peers. Overcrowded classrooms were also associated with a sense of low control, abusive dynamics, and obstacles to learning. These findings open the debate about safe spaces for girls, and other dissident identities in single-sex schools vs. mixed- gender spaces. On the one hand, traditional single-sex schools have been criticized for reinforcing stereotypical gender roles (53). On the other, not all students value mixed-gender spaces equally, particularly when these spaces contribute to experiences of violence or lack of protection. Such conditions can negatively affect self-esteem, academic performance, and the development of social skills. This highlights the importance of strengthening coeducational models that promote gender equity both inside and outside the classroom, while also preventing the reinforcement of structural inequalities historically experienced by girls and adolescent females (54). This highlights the importance of navigating identity, the systemic barriers faced by girls and non-binary people, and the psychological impact of these intersectional forms of oppression (55).

Finally, we want to highlight three key reflections that emerged from the collective discussions. The first concerns differences in students' emotional well-being based on their country or family background, both within and beyond the educational sphere. Although no explicitly discriminatory practices were reported at school, students perceived a cultural clash and felt differences between their own backgrounds and the norms of the host country, particularly in relation to the neighborhood and Chilean society. Unpleasant feelings emerged, including emotions such as fear and shame, mainly due to differences in accents and cultural practices. These differences contributed to greater social isolation and difficulties in experiencing a sense of belonging. These results align with research on Emotional Geographies, which validates the experience of nostalgia and the importance of the country of origin as a place of emotional well-being and idealized belonging (56). This underscores the need for the school system to foster welcoming and supportive environments for migrant students, while also creating opportunities for mutual understanding among all students. In other words, it calls for the promotion of critical intercultural education—one that increases educational opportunities for all and acknowledges multiple worldviews and gender identities (57). These findings underscore the need to provide personalized support services for non-binary people from an intersectional perspective in the areas of health, education, and community support. They also advocate for more comprehensive, culturally sensitive, and intersectional approaches to research and policy development (55). As a strong point, in this study adolescents emphasized the importance of shared spaces for coexistence in high school, festive activities celebrating the cultural heritage of students‘ countries of origin, and the value of having classmates from the same country.

The second reflection concerns the need for spaces for dialogue, as adolescents indicated that there were insufficient opportunities to share these or other reflections. They especially valued having time to be heard by adults external to the school community and without educational authority, which they described as an exceptional experience. This points to a critique of the prevailing adult centrism in educational settings, emphasizing the importance that dialogue spaces be mediated by adults who act as facilitators rather than authority figures imposing their worldview (58). This criticism is consistent with studies that have documented how pedagogical shortcomings and inattentive or abusive teacher dynamics are key factors that decrease satisfaction with school life (59). Adolescents stressed the need to recognize and value their autonomy, perspectives, and experiences, and to provide them with opportunities to actively participate in shaping dialogue and decision-making spaces. The need to recognize and value their particularities and autonomy aligns with the imperative to position adolescents as expert subjects whose experiences must be the starting point for understanding and socially constructing well-being (20). This reflection invites us to reconsider the role of adults not only in education but also within families, community, and health settings, to promote more equitable and collaborative intergenerational participation. Moreover, adolescents highlighted the value of the project as a space that allowed them to listen to one another, share experiences, find common ground, and recognize differences, which in turn fostered both social and emotional support.

Lastly, the use of non-traditional methodologies like Photovoice was especially when conducting research with adolescents, as it facilitated the expression of their experiences and emotions both visually and narratively. The images shared by participants communicated complex aspects and different emotional nuances that would be harder to express through traditional methodologies such as interviews, as they elicited discourse from a less conscious and more symbolic register. This visual approach aligns with adolescents' typical communication styles, for whom images are a more natural and accessible means of expression. Epistemologically, it recognizes the role of subjectivity in scientific research, as people not only narrate and share experiences or opinions but also engage in their analysis. Additionally, combining photographs with personal narratives promotes adolescents' agency, by giving them a meaningful voice in the research process, and fostering their participation, commitment, autonomy, and sense of ownership over their experiences. Likewise, the production of materials, such as the video and photo book, following data collection and analysis has allowed the results to endure over time and be shared locally and internationally, contributing to wider dissemination and impact beyond a scientific article.

Therefore, our findings highlight the need to engage intersectionality as a structurally anchored analytic framework rather than reducing it to a descriptive catalog of identity differences. Following foundational contributions to intersectionality that emphasize the co-constitution of power, social hierarchies, and lived experience (60), and aligning with the collective health paradigm's understanding of the social production of health, we argue that adolescents' emotional well-being must be interpreted within the broader historical and political-economic conditions shaping inequality in Chile. In this context, gender, racialization, and migratory background do not operate as discrete or additive identity markers; rather, they intersect with structural determinants such as urban segregation, socioeconomic precarity, and limited access to safe and supportive public spaces. The photovoice process illuminated how these overlapping axes materialize in the everyday experiences of adolescents living in intercultural neighborhoods, revealing how emotional distress, feelings of exclusion, and constrained agency emerge from cumulative and historically sedimented forms of marginalization. This structural approach also exposes conceptual tensions: while institutional and policy-driven categories tend to essentialize identities (e.g., “migrant youth,” “at-risk adolescent”), the adolescents' visual and narrative accounts reflect far more dynamic and relational forms of vulnerability and resilience. By grounding intersectionality in structural analysis, rather than in identity alone, our study strengthens the understanding of adolescent emotional health as shaped by unequal social relations and territorialized forms of injustice characteristic of contemporary Chile.

Finally, the main limitation of the study was the limited time and resources available to conduct a long-term follow-up of the entire process and to collaborate more actively in dissemination and maximizing the potential for greater political impact with the results. A more longitudinal process would have been especially valuable to deepen the understanding of changes in emotional health through imagery and narrative during a stage characterized by many transformations. We believe it is important to report on these types of studies and to highlight the need to challenge adult-centric development models in education, health, and society.

## Conclusions

Building on these findings, our analysis reinforces the need to understand adolescents' emotional well-being through a structural intersectionality lens that explicitly includes gender diversity, encompassing non-binary and gender-expansive youth. The ways in which solitude, peer support, family leisure, activism, and relationships with nature are experienced cannot be disentangled from the broader social conditions that shape adolescents' lives in Chile. Gendered expectations regarding care and domestic labor, the racialization and culturalization of migrant and diasporic groups, and persistent socioeconomic precarity intersect to delimit the time, space, and institutional support available for emotional flourishing. While friendships, companion animals, and activist spaces provided recognition and belonging, these protective factors emerged alongside material constraints—limited family time due to labor demands, territorial insecurity, and the unequal distribution of green and safe public spaces. Concerns about environmental degradation and the climate crisis further illustrate how emotional well-being is embedded in ecological and political-economic processes, resonating with collective health approaches that conceptualize subjectivity as socially produced. For migrant, intercultural, and gender-diverse adolescents, the absence of institutional recognition amplified feelings of isolation, highlighting how schools and community institutions can both mediate access to resources and reproduce exclusionary dynamics. Taken together, these findings call for educational, health, and community models that move beyond identity-based inclusion and address intersecting structural determinants, while recognizing adolescents of diverse identities as active agents in shaping the policies and interventions that affect them.

Based on these situated findings, we offer specific recommendations to enhance emotional health within the educational environment. The results suggest that schools can serve as pivotal spaces for well-being by moving beyond academic instruction to foster community care. This involves creating non-hierarchical listening spaces where adolescents can express themselves without adult-centric judgment. Furthermore, preserving green areas and free time for autonomous peer socialization is essential, as these interactions emerge as key protective factors. Finally, we suggest that schools actively recognize student diversity and significant affective bonds, including the role of companion animals—as valid components of their support networks. These measures would allow educational institutions to address emotional distress not in isolation, but by strengthening the relational and collective fabric of the school community.

Future research should further examine how gender diversity—including the experiences of non-binary and gender-expansive adolescents—intersects with racialization, migratory background, and socioeconomic conditions to shape emotional well-being across different territories in Chile. It is also important to explore how environmental degradation, access to green and safe public spaces, and perceptions of climate uncertainty influence adolescents' affective experiences. Further studies should investigate how institutional practices in schools and communities either reinforce or mitigate exclusion among migrant, intercultural, and gender-diverse youth. In addition, understanding how the social organization of care, family time constraints, and different forms of relational support—friendship, activism, human–animal bonds, and peer networks—contribute to emotional health remains an essential area of inquiry. Finally, future work could advance participatory and creative methodologies, such as through artistic and activist research or ethnography to better capture the lived experiences of adolescents with diverse identities and to position them as co-producers of knowledge within collective health research. In this way, we propose perspectives from adolescents that do not homogenize poverty, gender, or migration.

## Data Availability

Data cannot be shared publicly because of ethical restrictions. The Ethical Committee does not allow us to share the data publicly as our data contain sensitive personal information and cannot be fully anonymized. Our study has been approved by the Research Ethics Committee of the Faculty of Social Sciences at the University of Chile (Reference N° 45-60/2022). For more information on data availability restrictions you can contact the ethics committee at comite.etica@facso.cl.
